# Asthma is a risk factor for acute chest syndrome and cerebral vascular accidents in children with sickle cell disease

**DOI:** 10.1186/1476-7961-3-2

**Published:** 2005-01-21

**Authors:** Mark E Nordness, John Lynn, Michael C Zacharisen, Paul J Scott, Kevin J Kelly

**Affiliations:** 1 Department of Pediatrics, Medical College of Wisconsin, Milwaukee, Wisconsin, USA; 2 Prohealth Care Medical Center, N17 W24100 Riverwood Drive, Suite 150, Waukesha, Wisconsin, 53188, USA; 3 Department of Pediatrics, Medical College of Wisconsin, Milwaukee, Wisconsin, USA; 4 Department of Pediatrics, Children's Research Institute, Medical College of Wisconsin, Milwaukee, Wisconsin, USA; 5 Department of Pediatrics, Children's Research Institute, Medical College of Wisconsin, Blood Center of Southeastern Wisconsin, Milwaukee, Wisconsin, USA; 6 Department of Pediatrics, Children's Research Institute, Medical College of Wisconsin, Milwaukee, Wisconsin, USA

## Abstract

**Background:**

Asthma and sickle cell disease are common conditions that both may result in pulmonary complications. We hypothesized that children with sickle cell disease with concomitant asthma have an increased incidence of vaso-occlusive crises that are complicated by episodes of acute chest syndrome.

**Methods:**

A 5-year retrospective chart analysis was performed investigating 48 children ages 3–18 years with asthma and sickle cell disease and 48 children with sickle cell disease alone. Children were matched for age, gender, and type of sickle cell defect. Hospital admissions were recorded for acute chest syndrome, cerebral vascular accident, vaso-occlusive pain crises, and blood transfusions (total, exchange and chronic). Mann-Whitney test and Chi square analysis were used to assess differences between the groups.

**Results:**

Children with sickle cell disease and asthma had significantly more episodes of acute chest syndrome (p = 0.03) and cerebral vascular accidents (p = 0.05) compared to children with sickle cell disease without asthma. As expected, these children received more total blood transfusions (p = 0.01) and chronic transfusions (p = 0.04). Admissions for vasoocclusive pain crises and exchange transfusions were not statistically different between cases and controls. SS disease is more severe than SC disease.

**Conclusions:**

Children with concomitant asthma and sickle cell disease have increased episodes of acute chest syndrome, cerebral vascular accidents and the need for blood transfusions. Whether aggressive asthma therapy can reduce these complications in this subset of children is unknown and requires further studies.

## Background

Sickle cell disease is a common debilitating hematologic disease occurring in 1 in 650 African Americans. Lung disease is a major cause of cardiopulmonary disability and mortality [1,2]. Progressive restrictive lung disease related to recurrent episodes of acute chest syndrome may develop with advancing age [3]. Acute chest syndrome is a clinical manifestation triggered by pathological processes including infections, fat embolism, infarction and bronchospasm [4]. Nearly 70% of patients with acute chest syndrome are hypoxemic (as measured by pulse oximetry < 90% or PO_2 _< 80 mmHg by blood gas analysis) [5]. Hypoxemia causes sickle hemoglobin to gel, inducing red blood cell sickling and vaso-occlusion within pulmonary blood vessels.

Asthma is the most common chronic disease of childhood occurring in 6% of the general population. Moreover, the African American population has the highest prevalence (8%), emergency department (ED) visits, hospitalizations and risk of mortality of any ethnic population in the United States [6]. Reversible airway obstruction, airway inflammation and nonspecific bronchial hyperresponsiveness are the hallmarks of asthma. Exacerbations of asthma result in mucous plugging, bronchoconstriction with decreased air exchange, ventilation-perfusion mismatch and subsequently hypoxemia. Aggressive treatment with oxygen, bronchodilators and oral corticosteroids are recommended for symptomatic relief of acute episodes.

The African American population is at significant risk for the occurrence of both diseases simultaneously but little is known of the affect of asthma on individuals with sickle cell anemia. In this study, we examined hospitalized children with sickle cell disease with concomitant asthma establishing whether there was an increased rate of acute chest syndrome or other complications compared to patients with sickle cell disease without asthma in those presenting with vaso-occlusive pain crises.

## Methods

We performed a 5-year retrospective chart review of 48 children with asthma and sickle cell disease and compared them to a control group of 48 children with sickle cell disease alone. The 48 children with sickle cell and asthma represented all patients with complete medical records with both diseases that were cared for at our center. These children had no hospitalizations for sickle cell crises at other facilities. Children in the control group were matched for age, hemoglobinopathy and gender. Sickle cell disease was determined by high performance liquid chromatography and isoelectric focusing analysis and grouped into phenotypes of hemoglobin SS or hemoglobin SC.

Children were included in the asthma group if they had medical record documentation of a discharge diagnosis of asthma (ICD-493) and had been prescribed asthma medications.

Vaso-occlusive pain was defined as pain that could not be explained by injury or infection requiring hospital admission and treatment with intravenous pain medication. The criteria for a diagnosis of acute chest syndrome included respiratory distress, hypoxemia, and a new infiltrate on chest x-ray that required hospitalization and a transfusion of packed red blood cells. A cerebral vascular accident diagnosis was based on the new onset of an acute neurological syndrome with a focal neurological finding on examination associated with ischemic changes (images compatible with stroke) on a brain MRI or computed tomography scan.

Inpatient admissions were recorded from March 1997 to March 2002 for episodes of acute chest syndrome, vasoocclusive pain crises, cerebral vascular accidents, total blood transfusions, exchange transfusions and chronic transfusions (monthly blood transfusions).

Exclusion criteria included any child who had incomplete documentation of their hospital records or those who had moved into or out of the Milwaukee area during the 5-year study period. Six children were excluded because they moved from Milwaukee. The study was approved by the Investigational Review Board.

### Statistical Methods

The Mann-Whitney test was used to evaluate differences between the incidents of vasoocclusive pain crises, acute chest syndrome, total blood transfusions and cerebral vascular accidents. Chi-squared analysis was used to evaluate the number of exchange and chronic transfusions. Statistical significance was given to p values 0.05 or less.

## Results

All subjects in the asthma group had asthma recorded in their medical record with symptoms consistent with asthma. All had been prescribed albuterol; while 28 (58%) had been prescribed controller medications (24 inhaled corticosteroids, 12 inhaled cromolyn sodium, and 5 leukotriene modifier). Most patients had been prescribed combination therapy with more than 1 controller medication or had been switched from 1 controller to another. There was no reliable means to confirm adherence to the prescribed asthma drug regimen. Seventeen (35%) subjects had not had prior ED or hospital admissions for asthma. Of these children, 13 (76%) had been prescribed only albuterol. Twelve (25%) had at least 1 ED visit and at least 1 hospital admission for asthma. Twenty-six (54%) subjects had only hospital admissions for asthma, while 15 children had only been treated in the ED for acute asthma. Of 14 children with a severity category documented, 9 had mild intermittent asthma, 1 each with mild persistent and moderate persistent asthma, and 3 with severe persistent asthma. No patient had documented bronchoprovocation with methacholine and only 3 had documented spirometry with 1 consistent with airway obstruction, 1 with reversibility of airway obstruction, and one with poor technique and unreliable results. Two patients (age 3) were too young for spirometry. Four subjects had been seen in the Asthma and Allergy clinic for consultation and the diagnosis of asthma reaffirmed. Twenty-one (44%) subjects had a primary family member with asthma. Only 5 children were born preterm (27, 33, 34, 36, and 37 weeks gestation) and none were diagnosed with bronchopulmonary dysplasia.

The cases and controls were well-matched for age, gender and type of hemoglobinopathy (Table I). The cases (21 males and 27 females) consisted of 42 children with HgbSS and 6 with HgbSC. The control group (17 males and 31 females) included 41 children with HgbSS and 7 with HgbSC. The age of the children ranged from 3 to 18 years old with a mean age of 10.1 years (median 10 years) for the case patients and 10.3 years (median 10 years) for the control children.

**Table 1:** Subject Demographics

	**Patients with Sickle Cell & Asthma**	**Patients with Sickle Cell Disease**
**Males**	21	17
**Females**	27	31
**Mean Age**	10.1 years	10.3 years
**HgbSS**	42	41
**HgbSC**	6	7

Patients with sickle cell disease and asthma had significantly more episodes of acute chest syndrome, cerebral vascular accidents, blood transfusions and chronic transfusions as compared to the control group (Table 2). Admissions for vaso-occlusive pain crisis and exchange transfusions were not statistically significant between groups.

**Table 2:** Results in children with sickle cell disease with and without asthma

	**Patients with Sickle Cell & Asthma (n = 48)**	**Patients with Sickle Cell Disease (n = 48)**	**Statistical Significance**
**Admissions for Acute Chest Syndrome**	90	58	P = 0.03
**Number of Cerebral Vascular Accidents**	10	2	P = 0.05
**Total Blood Transfusions**	432	226	P = 0.01
**Chronic Blood Transfusions**	13	4	P = 0.04
**Vaso-occlusive Pain Crises**	248	223	P = 0.52
**Exchange Blood Transfusions**	9	4	P = 0.21

No patient with SC disease in either group had a history of a cerebral vascular accident. Only 1 patient with SC disease had acute chest syndrome, while 3 children in the asthma group had acute chest syndrome with one child experiencing 2 episodes.

## Discussion

Despite the high prevalence of these two diseases that affect the African American population, there is paucity of research investigating patients with concomitant asthma and sickle cell disease.

In this study, we discovered that children with both asthma and sickle cell disease are significantly more likely to develop severe complications of sickle cell disease including acute chest syndrome and cerebral vascular accidents compared to children with sickle cell disease alone. The significance of these findings relates to the hypothesis that appropriately aggressive treatment of asthma in children with sickle cell disease may diminish the frequency of pulmonary complications.

Patients with reactive airway disease and sickle cell disease have a lower transcutaneous oxyhemoglobin saturation [7]. The lower transcutaneous oxyhemoglobin saturation increases sickling of red blood cells, causing subsequent gelling and vaso-occlusion in multiple organs leading to numerous complications. A high prevalence (73%) of airway hyperreactivity to cold-air challenge occurs in children with sickle cell anemia even in the absence of clinical symptoms of asthma [8]. Cold air or other provocative challenge tests had not been performed in our group of patients. Our results are in agreement with an abstract that showed 18 children with both asthma and sickle cell disease had increased hospital admissions for pain crises and acute chest episodes compared with patients with only sickle cell disease [9]. Our results are unique because we assessed for an increased frequency of transfusions and cerebral vascular accidents in a larger group of patients.

Cerebral vascular accidents affect 10% of the children with sickle cell disease often with devastating long term implications. The Cooperative Study of Sickle Cell Disease reported that recent and recurrent episodes of acute chest syndrome are risk factors for cerebral vascular accidents [10]. Our findings of increased cerebral vascular accidents in patients with sickle cell disease and asthma may be due to their increased and recurrent episodes of acute chest syndrome. The mechanism linking stroke and acute chest syndrome is unknown but may be related to hypoxemia due to pulmonary disease. Hypoxemia has been reported to increase adhesion of red blood cells to endothelium [11]. Likewise, increased red blood cell adhesion to pulmonary endothelium has been associated with bone marrow vaso-occlusive crises due to hypoxia, cytokine expression and fat embolization [12]. Whether hypoxemia secondary to asthma affects red blood cell and pulmonary/cerebral endothelium adhesion characteristics is unknown.

Our study demonstrates that pediatric patients with sickle cell disease and asthma are more likely to require acute and chronic blood transfusions. It is not surprising that these patients needed more frequent transfusions since treatment for cerebral vascular accidents and severe acute chest syndrome is blood transfusions. Although SS type of sickle cell anemia is more severe than SC type, the small numbers of patients with SC prevent us from making broad conclusions regarding the degree of risk based on hemaglobinopathy.

Two studies have demonstrated increased airway hyperreactivity with reversibility in patients with sickle cell disease without known asthma [8,13]. Thirty-five percent of sickle cell patients had evidence of lower airway obstruction and 78% of these reversed with bronchodilator. Even in 30% of those with normal lung function, 30% had a positive response to bronchodilator. Therefore, even methods to determine the presence bronchial hyperresponsiveness are not sufficient to discriminate asthma from acute chest syndrome. Whether this hyperreactivity is due to asthma or is secondary to the pathophysiology of sickle cell disease is still unclear. Additional studies are needed to confirm that sickle cell disease is associated with the development of reversible airway obstruction. If the association is valid, the effects of routine use of anti-inflammatory controller agents prophylactically or therapeutically would deserve investigation.

A randomized, placebo-controlled trial of 43 episodes of acute chest syndrome in 38 children revealed that intravenous dexamethasone prevented clinical deterioration in mild to moderately severe episodes of acute chest syndrome [14]. Mild and moderately severe acute chest syndrome was defined as respiratory distress, and normal mental status without pulmonary infiltrates or arterial hypoxemia. The study excluded children with an exacerbation of reactive airways disease. If patients with sickle cell disease are at increased risk of airway inflammation and obstruction, successful treatment with intravenous steroids may have been due to aggressive treatment of underlying asthma.

Limitations of our study include those inherent in a retrospective analysis including selection bias, measurement bias and confounding factors. The children who received transfusions for acute chest syndrome likely represent the more severe form of disease. In contrast, those patients deemed to have asthma were primarily being treated as if they had mild to moderate asthma. Only 3 had been diagnosed with severe persistent asthma and none were on daily or every-other-day oral steroids. Importantly, even individuals with mild intermittent asthma can experience severe asthma exacerbations. Other sources of potential error that deserve recognition include the diagnosis of asthma (whether over-diagnosed or under-diagnosed), medication compliance and unknown admission to other hospitals. Although a strict definition of asthma was lacking, the history gleaned from the medical records supported an asthma diagnosis. Most patients with asthma are not cared for by asthma specialists and frequently the diagnosis is made on clinical grounds without formal pulmonary function testing. Additionally, spirometry was not routinely performed during ED and hospital admissions. Although other EDs and hospitals in the metropolitan area evaluate, treat, and admit pediatric patients with asthma exacerbations, the concomitant diagnosis of sickle cell disease likely prompted evaluation at the children's hospital.

## Conclusions

In this small series, children with a history of asthma and sickle cell disease developed acute chest syndrome and cerebral vascular accidents more frequently than children with sickle cell disease without asthma. The implications of this retrospective study are wide ranging and should lead to further prospective investigations. Whether aggressive asthma therapy in patients with sickle cell disease and asthma reduces the incidence of serious complications is unknown. The potential gains are far reaching and could make enormous impacts on the morbidity, quality of life and mortality of many patients.

## Competing interests

The author(s) declare that they have no competing interests.

## Authors' contributions

MEN conceived the study, participated in its design and coordination and drafted the manuscript. JL participated in data collection. MCZ participated in coordination and manuscript preparation. JPS participated in design, coordination and manuscript preparation. KJK participated in design and coordination.
